# Eco-Friendly Acaricidal Effects of Nylon 66 Nanofibers via Grafted Clove Bud Oil-Loaded Capsules on House Dust Mites

**DOI:** 10.3390/nano7070179

**Published:** 2017-07-10

**Authors:** Joo Ran Kim, Seong Hun Kim

**Affiliations:** Organic and Nano Engineering; College of Engineering, Hanyang University, Sung-dong-gu, Seoul 04763, Korea; jk992@cornell.edu

**Keywords:** clove oil, eugenol, nanofiber, microcapsules, house dust mites

## Abstract

Acaricidal nylon 66 fabrics (AN66Fs) grafted with clove oil-loaded microcapsules (COMCs) were developed against *Dermatophagoides farina* (*D. gallinae*). The average diameter was about 2.9 µm with a range of 100 nm–8.5 µm. COMCs carried clove oil loading of about 65 vol %. COMCs were chemically grafted to electrospun nylon nanofibers by the chemical reactions between –OH groups of COMCs and –COOH end groups of nylon fabrics to form ester linkages. AN66Fs had an effect on *D. farinae* depending on COMCs loadings. The increase in COMCs loading of AN66Fs from 5 to 15 wt % increased from 22% to 93% mortality against *D. farinae* within 72 h. However, AN66Fs containing over 20 wt % COMCs were more effective, showing up to 100% mortality within 24 h because the large amount of monoterpene alcohol, eugenol. This research suggests the use of clove oil and its major constituent eugenol as eco-friendly bioactive agents that can serve as a replacement for synthetic acaricides in controlling the population of *D. farinae*.

## 1. Introduction

Currently-used synthetic acaricides, such as the pyrethroid species, pose risks to human health when exposed to the environment, or humans; one such issue is neurotoxicity [1]. Synthetic pyrethroids, which perform on Na^+^ channels of the nerve cell membranes, can also have harmful effects on insects, such as honey bees [2]. In addition, these synthetic chemicals are hard to break down, so they build up quickly to a toxic point, which may present health risks to humans and the environment. Food quality protection acts recently restricted the sale of many commercial pyrethrum-based acaricides or pesticides [3]. This problem increases public pressure to provide safe or natural acaricides that have been produced in a more environmentally-friendly manner [3,4]. As a result, increased interest is now focused on more selective, natural compounds which are not toxic to humans and the environment, to reduce or eliminate reliance on synthetic acaricides or pesticides [5].

Natural essential oils are increasing in market demand due to their antimicrobial or acaricidal properties derived from the phenolic compounds of essential oils [6,7,8,9]. They have been studied as efficient, environmental-friendly, economic, and non-toxic acaricides to humans in the indoor environment [10,11,12]. The volatile properties of phenolic compounds, including alkanes, alcohols, aldehydes, and monoterpenoids displayed fumigant activities against a wide range of bacteria, fungi, and mites [13,14,15]. For example, essential oils, such as lavender, thyme, rosemary, marjoram, savory, and dillsun at 2 wt % and 1 wt % concentrations showed the mortality of greater than 97% and 95% on *Varroa* mites, respectively [16]. Additionally, 2 wt % spearmint demonstrated over 97% mortality on *Varroa* mites [16]. Cade, clove bud, coriander, horseradish, and mustard oils derived from plants at concentrations of 0.28 mg·cm^−2^ showed 99% mortality against the poultry red mite, *D. gallinae* [15]. Thyme and pennyroyal oils have also been shown as effective acaricides against *D. gallinae* [17]. The derivate from *Lauraceae* tree [18], phenolic compounds derived from *Chamaecyparis obtusa* leaves [19], 3-methylphenol isolated from *Ostericum koreanum* [20], and Perilla oil [21] was reported to show an effect on house dust mites. Pennyroyal oil composed principally more than 97% pulegone showed a mortality of more than 98% against *D. pteronyssinus* at 0.025 µL·cm^−2^ within a 5 min exposure time [22]. Furthermore, Kim et al. have studied the acaricidal effects of 56 natural plant essential oils on *D. gallinae* [15]. Among them, clove, thyme, horseradish, and coriander oils through direct contact and fumigation methods resulted in 100% mortality [15]. Additionally, other researchers reported that clove bud oil [6,23,24], thymol and cinnamaldehyde [25], and *Cnidium officinale* rhizome extracts [14] exhibited efficient mortality properties against *D. farinae* and *D. pteronyssinus.* Another example showed the major phenolic terpenoid components of essential oils, such as pulegone, α-terpinene, β-terpinene, eucalyptol, menthone, linalool, and fenchone had an effect of up to 100% mortality on *Tyrophagus putrescentiae* [26].

However, most essential oils are volatile or easily oxidized [3,27]. For the practical use of essential oils as antimicrobial, acaricidal, or pestisidal agents, it is necessary to improve convenient handling and stability by means of microencapsulation, which has been widely used for encapsulating essential oils [12,28,29]. The population of house dust mites and their allergens are predominantly found in non-washable bedding, pillows, and upholstery home textiles, such as carpets and sofas [30]. In order to reduce exposure to mite allergens, it is significant to develop anti-mite fabrics from non-toxic resources to humans and the environment. However, no one has studied the encapsulation of clove oil, and its grafting to nylon nanofibers to control house dust mites in the indoor environment.

In this study, acaricidal nylon 66 nanofabrics (AN66Fs) were developed from natural and environmentally-friendly resources with the minimum toxicity to humans and the environment to control the population of *D. farinae* (common indoor house dust mite species). The first step was to produce clove oil-loaded microcapsules (COMCs) by the coacervation method and to electrospin nylon 66 nanofabrics. Subsequently, COMCs were grafted to nylon 66 nanofabrics from 0 to 25 wt % of COMCs. The second step was to evaluate the acaricidal activity of the AN66Fs in terms of COMCs loadings against *D. farinae*.

## 2. Materials and Methods

### 2.1. Materials

Clove bud oil (CO, grade > 99%, density: 1.04 g·cm^−3^), Nile red dye, glutaraldehyde (GA, 25 wt % in water), poly(vinyl alcohol) (PVA, 35–50 kDa and 99 mol % degree of hydrolysis), anhydrous sodium sulfate, nylon 66 pellets, formic acid (reagent grade > 95%), and 4-(4,6-dimethoxy-1,3,5-triazin-2-yl)-4-methylmorpholinium chloride (DMTMM) were purchased from Sigma Aldrich Co. (Wonsam-myeon, Gyeonggi-do, South Korea). House dust mite (*Dermatophagoides farinae*) and the nutrient mixture for mites were donated from the School of Agricultural Biotechnology, Seoul National University (Seoul, South Korea).

### 2.2. Microencapsulation of COMCs by Oil-in-Water Simple Coacervation

**Figure 1:**
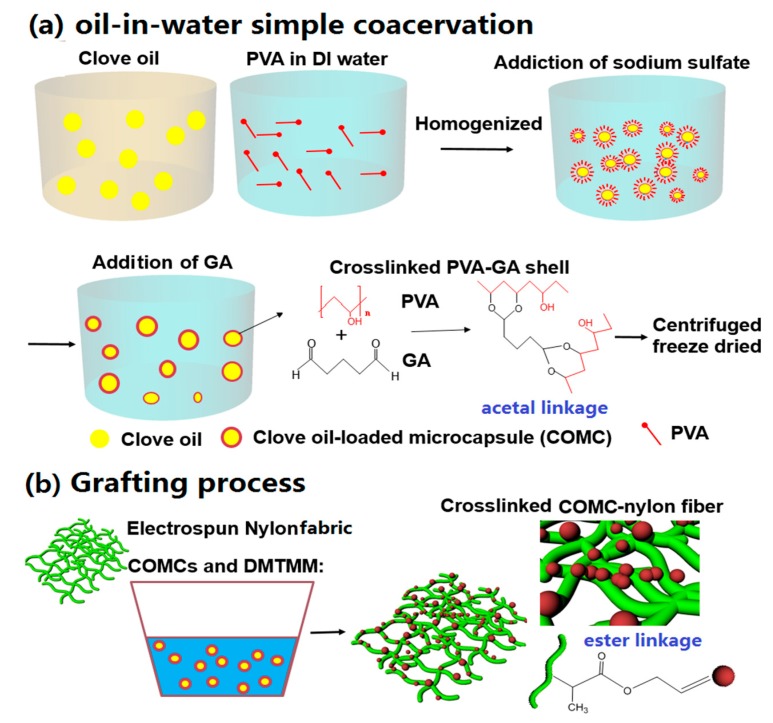
Scheme of the production of acaricidal nylon 66 nanofabrics (AN66Fs); (**a**) oil-in-water simple coacervation method to produce COMCs; and (**b**) the grafting procedure of nylon nanofibers with COMCs, the chemical reaction between carboxylic end groups of nylon 66 nanofibers and hydroxyl groups of PVA of COMCs form ester linkages.

A 3% (*w*/*v*) PVA was dissolved in 100 mL deionized (DI) water at 80 °C for 1 h and then cooled to room temperature (RT). CO (10 mL) was added to the PVA solution and homogenized using a homogenizer (Model-OV5, VELP Scientifica, Usmate, Italy) at 5000 rpm for 20 min to produce a CO-PVA emulsion. Sodium sulfate in DI water (15% (*w*/*v*)) was added to the CO-PVA emulsion and then homogenized at 5000 rpm for 10 min. A crosslinking agent, 2 wt % glutaraldehyde (GA) of PVA, was gradually added to the CO-PVA emulsion and agitated at 1000 rpm for 1 h above 50 °C. Figure 1a shows the scheme of the microencapsulation procedure of COMCs and the PVA-GA crosslinking reaction to form acetal linkages in the shell of COMCs [31,32]. Afterwards, microcapsules were collected using a centrifuge (Model: HA-1000-3, Daegeon, Korea) at 5000 rpm for 30 min and then washed with DI water containing 1 wt % ethanol a few times to eliminate any oil surrounding the COMCs and then freeze-dried for overnight.

### 2.3. Analysis of the Core Loading of COMCs

The CO loading within COMCs was quantified using a Clevenger-type apparatus. 10 g of freeze-dried COMCs was distilled for 3 h to isolate CO from COMCs while keeping the temperature above 250 °C. The evaporated CO was chilled by cold water in the condenser of a Clevenger-type apparatus. Cooled CO was weighed and calculated for the core CO loading, compared to the original weight of COMCs. The average core loading was calculated with three replications each for the three different samples.

### 2.4. The Internal Structure of COMCs

In order to observe whether COMCs contain CO as a core material and well-formed shells with a pore-free surface, one drop of Nile Red dye was added to CO, followed by the same microencapsulation procedure explained above in the experimental section. COMCs were observed using a confocal laser scanning microscope (CLSM; Leica TCS SL, Wetzlar, Germany) at 543 nm excitation wavelength, which was attached to 25 mW HeNe lasers with a 63× oil immersion lens (PlanApo 63× oil/1.40 NA/0.10 mm).

### 2.5. Nylon 66 Nanofiber-Based Fabrics (Nylon Nanofabrics) through Electrospinning

Nylon nanofabrics were produced using electrospinning. Initially, 10 wt % of nylon 66 pellets was dissolved into formic acid and stirred at 70 °C for 5 h on a hot plate. The cooled solution was added into a 5 mL syringe and then placed on the pump with a feeding rate of 500 µL per hour. Twelve kilovolts was applied between the needle tip and the collector. After 10 h, the randomly-oriented nylon nanofabrics were collected. A JEOL JSM-6340F field emission scanning electron microscope (FE-SEM, Hitachi, Japan) was used for observing the morphology of the nanofibers.

### 2.6. Grafting of COMCs onto Nylon 66 Nanofibers

The different loadings of COMCs at 0, 5, 10, 15, 20 and 25 wt % were suspended under stirring at 300 rpm in 10 mL of DI water, and electrospun nanofabrics (size dimension: approximately 10 × 10 × 0.2 cm) were immersed into each solution and DMTMM (2 wt % of COMCs) as a condensing agent was added to catalyze chemical reactions as shown in Figure 1b. The solution bath was stirred at 50 rpm for 6 h in order to complete further reaction. The crosslinking reactions occur between the hydroxyl groups of COMCs and carboxylic end groups of nylon 66 nanofibers, called the Fischer esterification reaction to form ester linkages chemically on nylon nanofibers [33,34,35]. Then the nanofabrics were removed from the solution. The AN66Fs were dried in oven for 12 h at RT. Fourier transform infrared spectroscopy (FTIR) equipped with a single-reflection attenuated total reflectance (ATR) system (Specac Ltd., London, UK) was used to characterize the surface chemical analysis of pure PVA, pure nylon 66 nanofabrics and AN66Fs with 15 wt % COMCs. Germanium was used for the ATR crystal (2 mm in diameter; depth of penetration: 0.65 µm; refractive index 4.0).

The specimens were scanned from 4000 to 600 cm^−1^ wavenumbers with a resolution of 2 cm^−1^. A total of 256 scans were taken to increase the signal/noise ratio. A thermogravimetric analyzer (TGA, PerkinElmer Pyris 1, Waltham, MA, USA) was used for the pure nylon 66, pure PVA, neat clove bud oil, and AN66Fs containing 5, 10, 15, 20 and 25% COMCs in the temperature range of 25–750 °C under a nitrogen flow rate of 100 mL·min^−1^.

### 2.7. The Acaricidal Effect of AN66Fs on D. farinae

The acaricidal effect of AN66Fs was evaluated against *D. farinae* according to the American Association of Textile Chemists and Colorists (AATCC) test method 194–2007, Assessment of Anti-House Dust Mite Properties of Textiles. In the first step, AN66Fs at different loadings of COMCs and the control were cut into circular shapes of 5 cm diameter. Cut AN66Fs and 50 mites were placed into the Petri dish with 10 cm diameter. 50 mg of nutrient mixture composed of albumin powder and dried yeast powder were put in each Petri dish. The edges of the Petri dishes were covered with a sticky gel and covered with micro-sized nylon mesh with a pore size under 50 µm to prevent mites from escaping. All specimens were kept at 25 °C and over 65% relative humidity. After 72 h treatment, the surviving *D. farinae* were counted under the optical microscope. All tests were conducted three times with three replicates. Dead HDM symptoms were characterized by immobility without walking or moving and shrunken legs. All specimens were counted at least twice. The following equation was used to determine the mortality of AN66Fs after 72 h:

(1) $$Mortality\;(\%)=\;\frac{x-y}{x}\times \;100$$

where *x* and *y* are the numbers of *D. farinae* found on the control specimen and the AN66Fs specimen after exposure time, respectively.

## 3. Results and Discussion

**Figure 2:**
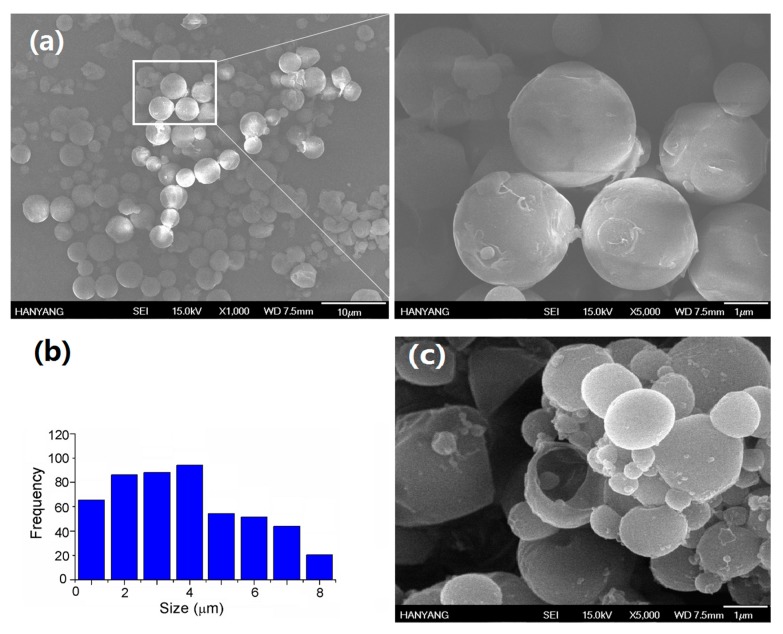
The size analysis of COMCs produced by the oil-in-water simple coacervation method; (**a**) the morphology of COMCs; (**b**) the size distribution histogram of COMCs; and (**c**) the broken COMC with shell thickness of 0.35 µm.

The morphology and size distribution of COMCs are shown in Figure 2. Under SEM observation, COMCs show spherical shapes and pore-free surfaces with a widespread size distribution. Figure 2b shows the diameter distribution histogram of COMCs measured from randomly-selected areas of SEM images (N ≥ 500). While the diameters of COMCs range between 0.2 and 8.5 µm, the average diameter is about 2.9 µm. COMCs demonstrated aggregation due to the uncrosslinked PVA residue present on the shell surface of COMCs [36]. The hydrocarbon part (CH_2_CH) of the PVA chains were possibly adsorbed onto the oil surface of COMCs, whereas the hydroxyl groups –OH) of PVA allowed the COMC microcapsules to adhere together to promote aggregation by hydrogen bonding. The broken COMCs have a diameter of 2.6 µm and shell thickness of approximately 0.35 µm, which presents about 65 vol % of clove oil (CO) as the core loading presented in Figure 2c.

**Figure 3:**
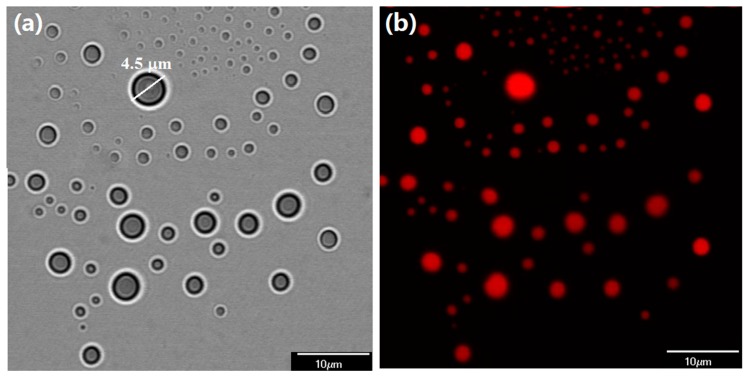
CLSM images showing the internal structure of COMCs; (**a**) a brightfield image; and (**b**) a fluorescent image (red color corresponds to clove oil stained with Nile red as the core loading).

Further evidence shows the internal structures of COMCs are obtained from CLSM. Figure 3 shows CLSM images showing the internal structures of COMCs. A brightfield image of COMCs confirms their spherical shape, free of voids with well-formed shells, ranging from a few hundred nanometers to a few micrometers, as shown in Figure 3a. For example, a COMC with a diameter of 4.5 µm and shell thickness of approximately 0.8 µm indicates that the core (clove oil) occupied about 65 vol % of the COMC. The results confirm the observations by SEM that COMCs have similar oil loading and a very wide size distribution. Figure 3b shows a fluorescence image of COMCs. The existence of clove oil stained with Nile Red is observed in red color. The oil loading of COMCs quantified by the distillation method was found to have 51 ± 4.8% by weight.

**Figure 4:**
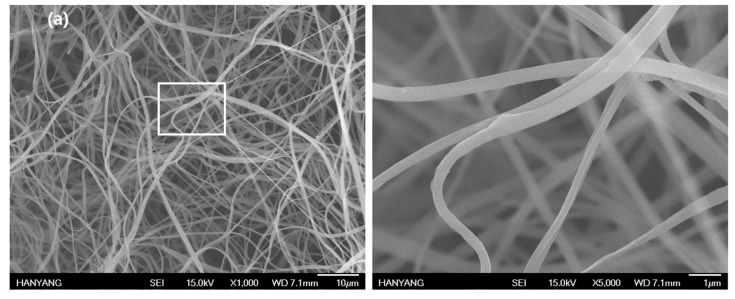

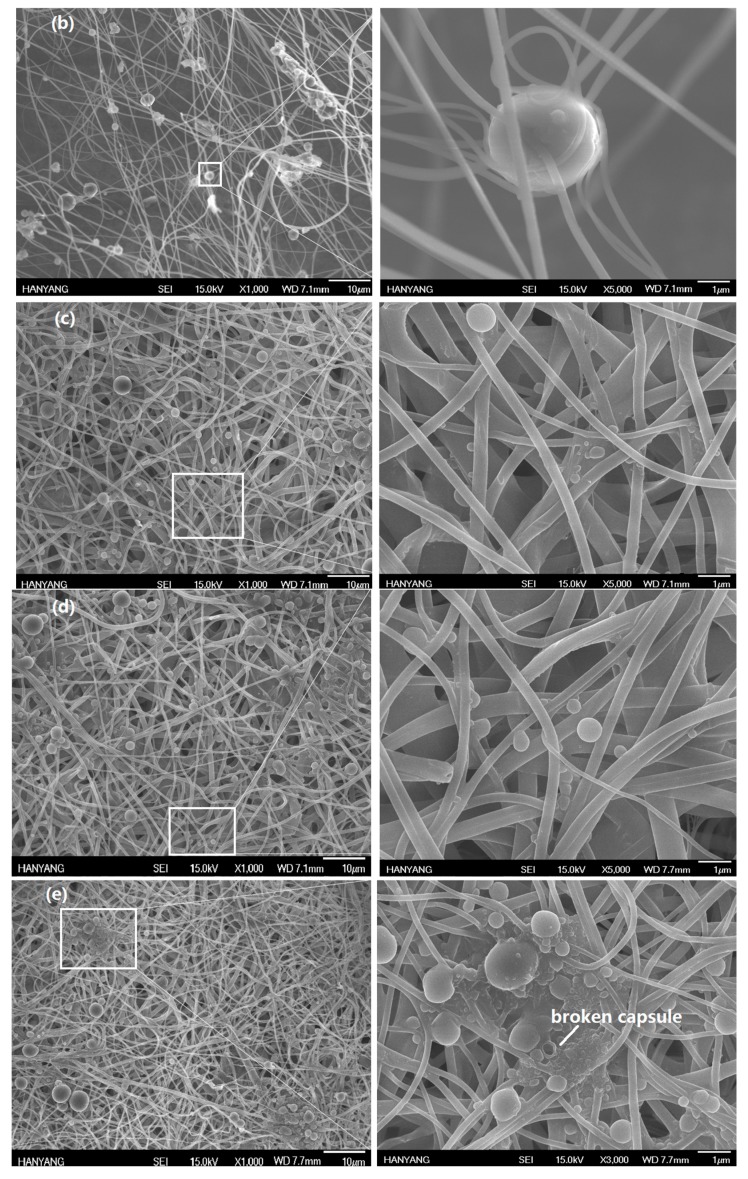

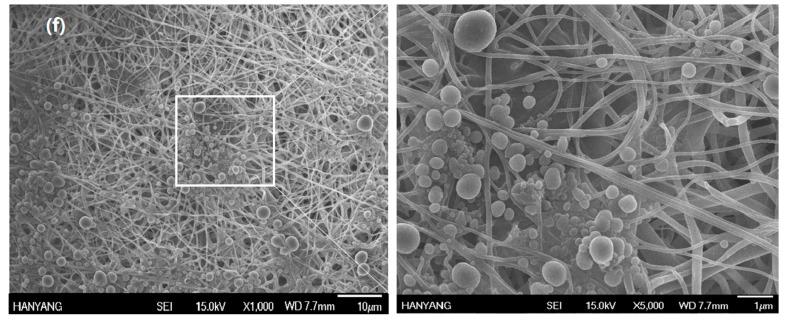
SEM images of AN66Fs at different loadings of COMCs; (**a**) 0 wt %; (**b**) 5 wt %; (**c**) 10 wt %; (**d**) 15 wt %; (**e**) 20 wt % and (**f**) 25 wt %.

After grafting COMCs to nanofabrics at different loadings of COMCs at 5, 10, 15, 20 and 25 wt %, the morphology of AN66Fs was observed by SEM. The diameters of nylon nanofibers range from 50 nm to 4.3 µm. Figure 4a–f show that COMCs are chemically attached to nylon nanofibers with increased loading of COMCs from 5 to 25 wt %. The chemical bonding on the nanofibers is because hydroxyl groups from unreacted PVA shells can crosslink chemically with carboxylic end groups on nylon nanofibers to produce ester linkages. These ester linkages increase the interfacial bonding between nylon nanofibers and COMCs. Hence, COMCs can break easily and allow the release of clove oil under friction and pressure of nanofabrics. Figure 4a shows neat electrospun nylon nanofibers without capsules. Figure 4b represents AN66Fs containing 5 wt % COMCs and displays the individual microcapsules attached to each nanofiber without aggregation of COMCs. Figure 4c,d show AN66Fs containing 10 wt % and 15 wt % of COMCs, respectively, and display well-dispersed COMCs on the nanofibers. However, the increasing COMC loadings over 15 wt %, increased the aggregates of COMCs on the nanofibers, as well as decreased the pores of AN66Fs, as shown in Figure 4d–f. There is a broken capsule which releases clove oil, as shown in Figure 4e. In the case of AN66Fs containing 20 wt % and 25 wt % COMCs, they show small pores due to the cluster of COMCs on the surface, and seem like coatings or films, as shown in Figure 4e,f.

**Figure 5:**
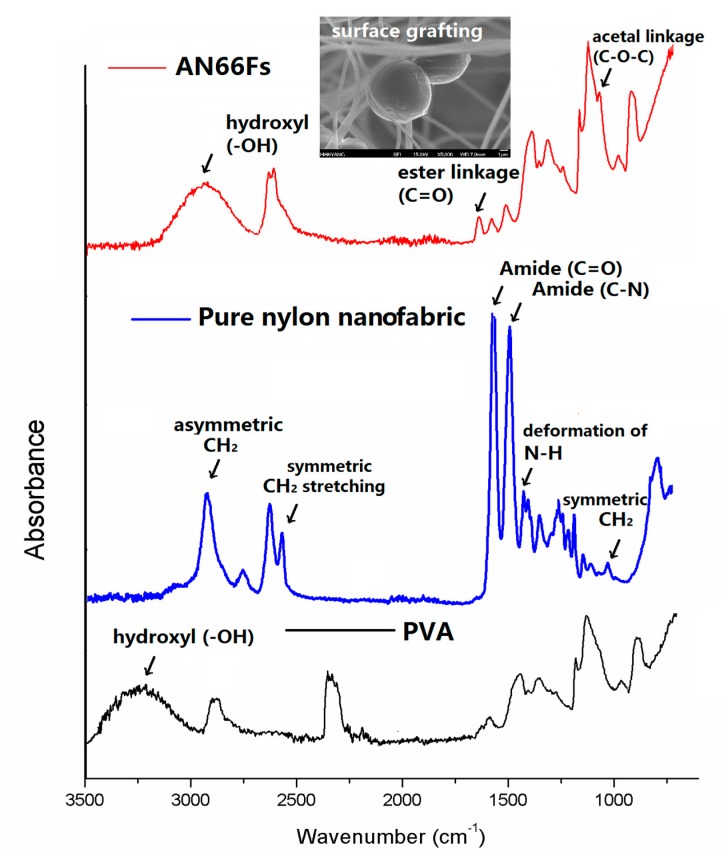
ATR-FTIR spectra of pure PVA, nylon 66 nanofabric, and AN66Fs.

Figure 5 shows ATR-FTIR spectra of pure PVA, pure nylon nanofabric and AN66Fs at 15 wt % COMCs. The FTIR spectrum of pure PVA shows the absorption associated with the C–H alkyl stretching band in the 2850–3000 cm^−1^ range. Strong hydroxyl groups for free hydroxyl (–OH stretching band) exists between 3000 and 3650 cm^−1^ [37]. Pure nylon nanofabric shows the absorption peaks at 2750–2980 cm^−1^ range (asymmetric C–H and CH_2_ stretching), 1120 cm^−1^, 2250 cm^−1^ and 2494 cm^−1^ (symmetric CH and CH_2_ stretching), and 1474 cm^−1^ (N–H deformation). Nylon 66 nanofabric has additional peaks at 1640 and 1554 cm^−1^ corresponding to amide groups, and C=O and C–N stretching from amide groups [38]. Similar IR absorption peaks have been observed by other researchers for nylon 66 fabrics [39]. In the case of AN66Fs, the crosslinked PVA–GA shell of COMCs shows the unique peak corresponding to C–O stretching at approximately 1135 cm^−1^, which can be attributed to the acetal linkages (C–O–C) [31]. The O–H stretching vibration peak in the range between 2600 and 3200 cm^−1^ was decreased, compared to pure PVA. The relative increase of the C=O band of ester linkages by the crosslinking reactions between –OH of PVA and –COOH end group of nylon 66 fibers at 1740 cm^−1^ was observed [31,37].

**Figure 6:**
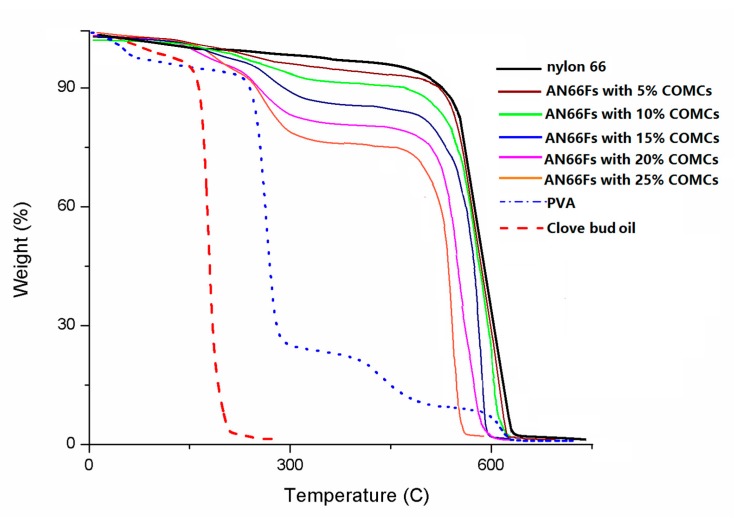
TGA thermograms of pure nylon 66, pure PVA, neat clove bud oil, and AN66Fs with 5, 10, 15, 20 and 25% COMCs.

Figure 6 shows TGA thermograms of pure nylon 66, pure PVA, neat clove bud oil and AN66Fs containing 5, 10, 15, 20 and 25% COMCs. TGA measures changes in the weight loss of AN66Fs containing different loadings of COMCs in the temperature range of 25–750 °C. Neat clove bud oil loses 100% of its weight when the temperature reaches 220 °C, while pure nylon 66 nanofabric undergoes thermal degradation beginning at 480 °C and finishing at 600 °C with a total mass loss of 99%. Pure PVA shows degradation points of 280 °C and 400 °C. The residual weight at 300 °C indicates the remaining nylon nanofabric and PVA since clove oil loses its weight completely at around 220 °C. From the results, higher residual weight at 300 °C matches less grafting loading of capsules onto nanofabrics. AN66Fs containing 25% COMCs show the highest weight reduction which indicates more capsules onto nanofabrics. AF66Fs with 5% COMCs show the lowest weight reduction, which means the fewest capsules grafting onto nanofabrics.

The mortality tests were conducted to evaluate the acaricidal effect of AN66Fs at different loadings from 0 to 25 wt % against *D. farinae*. All specimens were effective in reducing the number of *D. farinae* after 72 h treatment as shown in Table 1. In general, the mortality increased with an increase in COMCs loading to AN66Fs. The increase in COMCs loading to AN66Fs from 5 to 15 wt % greatly reduced the number of *D. farinae* from 22% to 93%. However, COMCs loading of over 20 wt % showed 100% mortality after 72 h. After 1 h of exposure time to AN66Fs with different loadings from 0 to 25 wt %, the number of surviving *D. farinae* resulted in less than 20% mortality. AN66Fs at 20 wt % and 25 wt % show nearly 100% mortality within 24 h, compared to AN66Fs (15 wt % COMCs loading) with only 72% mortality. This acaricidal activity is due to the hydrophobic property of clove oil, which plays a critical role in reducing the population of *D. farinae*. In addition, clove oil composed of more than 75% eugenol, which identified the phenolic monoterpenoid, exhibits powerful acaricidal activity [23,40]. The allyl group-derived eugenol may give stable non-ionic structure during the microencapsulation process [24,41]. Furthermore, clove oil is revealed as a plant-derived phenylpropanoid, which showed structural advantages in defense functions against microbial attack [42].

**Figure 7:**
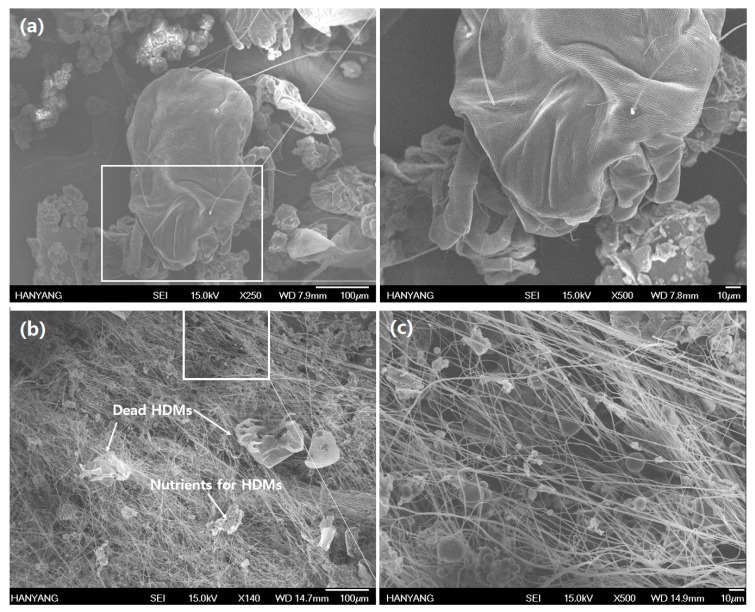
In vitro mortality tests of AN66Fs at 10 wt % COMCs after 72 h exposure of *D. farinae*; (**a**) the unpoisoned adult-sized *D. farinae*; (**b**) a knockdown-type of dead adult HDM with desiccation; and (**c**) aggregated COMCs on the nanofibers.

In Figure 7a, the unpoisoned adult-sized *D. farinae* is shown in the range of 200 to 300 µm in length. Figure 7b shows the poisoning symptom of *D. farinae* from treated fabrics with COMCs. It shows a knockdown-type death with desiccation on the body of *D. farinae*. Similar results were reported by Ignatowicz et al. and Kim et al. that dead *T. putrescentiae* by monoterpenoids showed depression of the dorsal surface as an idiosoma symptom related to be desiccation after shriveling, with legs folded under their bodies [43,44]. However, synthetic insecticides, such as pyrethroids, showed uncoordinated behavior or hollow skin surfaces after treatment [23]. In the present study, *D. farinae* showed forward leg movement and desiccation symptoms as their unique initial non-toxic signs induced by natural monoterpenoid resources. The results demonstrate that clove bud oil compounds possess acaricidal activityies by vapor action when COMCs break and release the bioactive (clove oil) on the fabrics. Eugenol, as the monoterpenoid containing hydroxyl groups in its structure, allows for greater water release from the body of *D. farinae*, thus supporting greater acaricidal activity resulting from desiccation [26]. The nanofabric grafted with 10 wt % COMCs shows 64% mortality. This finding may be attributed to insufficient loading of COMCs to inhibit *D. farinae*. A major reason for low mortality (64%) in nanofabrics with 10 wt % COMCs loading is that the COMCs remained unbroken due to their aggregated shape, as shown in Figure 7c. In addition, the low grafting yield of COMCs onto nanofabrics may limit the acaricidal effect on *D. farinae* due to limited chemical bonding sites between nylon 66 nanofibers and COMCs.

## 4. Conclusions

In this study, nanofabrics grafted with COMCs showed excellent acaricidal activity and the possibility to reduce allergen levels and clinical symptoms of house dust mite allergy in the household environment. Our results showed that the diameters of COMCs range between 0.1 and 8.5 µm, with the average diameter equaling 2.9 µm. The core loading (CO) was found to have about 65 vol %. When incorporated into textiles, such as beddings, home and medical textiles, the nanofabrics grafted with COMCs could be efficient acaricidal agents to reduce the population of *D. farinae*. The release of clove oil from COMCs had an effect on *D. farinae* because of eugenol, a stable allyl-derived structure, and served as a natural acaricidal agent. The increase in COMCs loading to nanofabrics from 5 to 15 wt % effectively reduced the number of *D. farinae* from 22% to 93%. However, COMC loadings of over 20 wt % showed 100% mortality within 24 h. This research presents the use of clove oil through a microencapsulation technique and grafting procedure on nanofabrics, showing practical usage in real life, therefore, serving as the replacement for synthetic or conventional acaricides. Due to its low toxicity, nylon 66 nanofabrics grafted with COMCs pose little risk to humans and the environment.

## Figures and Tables

**Table 1:** In vitro mortality tests of AN66Fs at 0, 5, 10, 15, 20 and 25 wt % COMCs loadings against *D. farinae* after 72 h with three replications.

COMCs Loading to Nylon Nanofabrics	0 wt %	5 wt %	10 wt %	15 wt %	20 wt %	25 wt %
Surviving Number of *D. farinae* after 72 h	50 (0.2)	39 (1.6)	18 (2.2)	4 (0.8)	1 (0.5)	0 (0)
Mortality (%)	0.1 (0)	22 (1.8)	64 (2.5)	93 (2.4)	100 (0.5)	100 (0)

Values in parentheses represent standard deviation.
